# Gastric Collision Tumors: An Insight into Their Origin and Clinical Significance

**DOI:** 10.1155/2015/314158

**Published:** 2015-02-12

**Authors:** Adamantios Michalinos, Anastasia Constantinidou, Michael Kontos

**Affiliations:** ^1^1st Department of Surgery, University of Athens, Laiko Hospital, 17 Agiou Thoma Street, 11527 Athens, Greece; ^2^The Institute of Cancer Research and the Royal Marsden Hospital, 15 Cotswold Road, London SM2 5NG, UK

## Abstract

Collision tumors are rare neoplasms displaying two distinct cell populations developing in juxtaposition to one another without areas of intermingling. They are rare entities with only 63 cases described in English literature. Tumors encountered are gastric adenocarcinomas colliding with lymphomas, gastrointestinal stromal tumors, squamous cell carcinomas, and neuroendocrine tumors. Their cell origin is obsolete by the time of diagnosis. Different tumorigenesis theories have been suggested to explain their behavior, yet none has managed to provide satisfactory explanation for all cases. Clinically they are indistinguishable from the dominant tumor. Lack of data does not allow detailed assessment of their behavior yet they seem aggressive neoplasms with dismal prognosis. The majority of cases have been diagnosed postoperatively during histologic examination of specimens. There are no guidelines or concrete evidence to support best way of adjuvant or other types of treatment. However, these rare neoplasms might help in unlocking secrets of cancer behavior including tumorigenesis, differentiation, and adhesion and thus clinicians should be aware of their existence.

## 1. Introduction

Gastric collision tumors are neoplasms consisting of two distinct cell populations, developing in juxtaposition to one another, without areas of intermingling [1–6]. They are rare tumors and are diagnosed only rarely preoperatively. Literature is comprised of case reports and a few case series; thus our knowledge on them is scattered. Their clinical and pathological behavior remains largely unknown as do appropriate diagnostic and therapeutic procedures.

Gastric collision tumors are usually comprised of an epithelial and a sarcomatous element. They should be differentiated from other entities like carcinosarcomas (a single neoplasm exhibiting a carcinomatous and a sarcomatous pattern), composite tumors (two different histological patterns intermingling in a single tumor), or cancer-to-cancer metastasis (carcinoma metastasizing to a different carcinoma) [7]. Neoplasms with two distinct populations but without a clear-cut interface between histological patterns or a transition zone of mixed character in between should be categorized as composite neoplasms rather than collision tumors [2, 8].

The first gastric collision tumor was probably described by Jernstrom and Murray in 1966 [9] and consisted of a gastric carcinoid colliding with an adenocarcinoma. Since then only 18 similar cases have been described in the literature (Table 1). Other types of collision tumors are lymphomas colliding with gastric adenocarcinomas (26 cases, Table 2), gastrointestinal stromal tumors (GISTs) colliding with gastric adenocarcinomas (9 cases, Table 3), squamous cell carcinomas colliding with gastric adenocarcinomas (7 cases, Table 4), and a few other isolated cases. Each category presents unique characteristics, different behavior, and probably different origin mechanism.

In this review authors present the published experience in gastric collision tumors. We study their clinical behavior and appropriate diagnostic and therapeutic procedures and review tumorigenesis theories.

## 2. Materials and Methods

An electronic bibliographic search was conducted in Medline Embase, Cinahl, and Cochrane Library for studies on gastric collision tumors. Terms used were “gastric collision tumor,” “neuroendocrine tumor and adenocarcinoma,” “lymphoma and adenocarcinoma,” “squamous cell carcinoma and adenocarcinoma,” and “gastrointestinal stromal tumor and adenocarcinoma.” Results were hand-searched and selected appropriately. Moreover, literature of selected articles was further hand-searched for relevant publications. Only articles in English were included in this analysis.

### 2.1. Neuroendocrine Carcinomas and Gastric Adenocarcinomas

To our knowledge, 18 cases of collision tumors between neuroendocrine carcinomas and gastric adenocarcinomas have been described to date, 14 in males and 4 in females (Table 1). The mean age of the patients was 61 years (47–84). The majority were nonfunctional carcinoids while large cell endocrine carcinoma [10] and gastrinomas [11, 12] have also been described. The latter were discovered during investigation for Zollinger-Ellison syndrome. Five of the gastric adenocarcinomas were poorly differentiated, 5 moderately differentiated, and 3 well differentiated. Most authors do not refer to lymph node status; however in 4 of the cases they were infiltrated by gastric adenocarcinoma, 3 by neuroendocrine tumor and in one from collision-type tumor.

### 2.2. Lymphomas and Gastric Adenocarcinomas

Collision between lymphoma and gastric adenocarcinoma is the most common category with 26 cases described to date. Eighteen were described in males and 8 in females. The mean age was 60 years (42–80). The majority of cases were MALT lymphomas.

Collision tumors between lymphomas and gastric adenocarcinomas present some interesting characteristics. According to Nishino et al. [13], lymphomas are usually larger than gastric adenocarcinomas and gastric adenocarcinomas are usually early and well differentiated; however, Nakamura et al. [14] reported that lymphomas are usually mucosa MALT lymphomas and gastric adenocarcinomas have higher Ki67 index. Prognosis is usually determined by the adenocarcinoma component. Goteri et al. [6] stated that glandular epithelium inside a MALT lymphoma might be more prone to neoplastic transformation due to continuous inflammation.

In total, of the published cases 9 were early gastric cancers and only 5 were advanced. Fourteen cases were well, 1 moderately, and 7 poorly differentiated. Eleven cases were of intestinal type, 5 of diffuse, and only 1 signet-ring cell carcinoma.

One case of collision between Hodgkin's lymphoma and Gastric adenocarcinoma has been also described [15]. This case is of particular interest since Hodgkin's lymphoma was also found in the perigastric lymph nodes. Authors believe that the lymphoma component metastasized to the stomach where it collided with a gastric adenocarcinoma.

### 2.3. Squamous Cell Carcinomas and Gastric Adenocarcinomas

Only 9 cases of collision between squamous cell carcinoma (SCC) and gastric adenocarcinoma have been described to date. Most cases were located at the gastroesophageal junction with SCC lying at the esophageal side and adenocarcinoma at the gastric side. All patients were males with mean age of 49 years (37–73). In a composite tumor described by Dodge [2] an anaplastic carcinoma was at the gastric side and adenocarcinoma at the esophageal side.

Milne et al. [5] formulated criteria for collision tumors of the gastroesophageal junction: (1) the two components should show topographical separation; (2) the squamous component should lie on the esophageal side of the tumor and the adenocarcinoma component on the gastric side; and (3) there should be little or no evidence of intermediate histologic structure in between.

### 2.4. Gastrointestinal Stromal Tumors and Gastric Adenocarcinoma

Only 7 cases of collision between gastrointestinal stromal tumor and gastric adenocarcinoma have been described. Four were males and 3 were females. Mean age was 72 years (54–86). In all these cases, GISTs were of low malignant potential while gastric adenocarcinoma was usually advanced.

Kleist et al. [16] described a rare case of a gastric adenocarcinoma inside a GIST. They reported that this could have occurred from dysplastic epithelium trapped inside GIST sustaining tumorigenic effect of in-tumor microenvironment or tumor-to-tumor metastasis from an independent gastric adenocarcinoma.

### 2.5. Other Cases

Finally, three unique cases have also been described in the literature. Dodge [2] in 1961 described a case of anaplastic carcinoma colliding with gastric adenocarcinoma, Adhikari et al. [17] in 2006 described a unique case of gastric angiosarcoma colliding with GIST, and Go [18] a case of schwannoma colliding with GIST.

### 2.6. Tumorigenesis Theories

The cell origin of collision tumors is obsolete by the time of diagnosis. Theories formulated for their origin, while valuable, are not satisfactory for all cases.

The oldest and most simplistic theory, adopted by older reports, is that of accidental meeting of two coexisting neoplasms developing independently and finally colliding [1, 9, 19, 20]. This theory does not provide any particular explanation of the colliding pattern and makes no differentiation between composite and collision neoplasms. Experimental studies in mice have shown that tumors developing in tumor environment display a more aggressive behavior, including infiltration characteristics [19]. This is also the case in rare tumor-to-tumor metastases cases [6, 19]. This explanation is challenged by other, modern theories and also by few studies providing molecular analysis of the tumor elements of collision tumors [5, 21] that have shown a single cell origin neoplasm. Still, since synchronous but remote gastric tumors have been described in approximately 1.25% of all gastric cancer cases [22], this theory cannot be completely refuted.

Common carcinogen or field theory suggests that a single carcinogen leads to development of two synchronous, colliding neoplasms. This theory is appealing for lymphoma-gastric adenocarcinoma collision tumors, especially since this carcinogen has probably been isolated.* H. pylori* is known to induce both lymphoma and gastric adenocarcinoma development.* H. pylori* is found in 45–90% of gastric adenocarcinoma patients and in 56% of lymphoma patients [14]. Suenaga et al. [23] believe that* H. pylori* infection is more common in those with synchronous lymphoma and gastric adenocarcinoma than with lymphoma or gastric adenocarcinoma alone.

Gastric adenocarcinoma promotion is probably attributed to chronic inflammation with cytokines, ammonia, and proteolytic enzymes involved in the process [6, 24]. Acute gastritis caused from* H. pylori* develops into chronic gastritis, atrophic gastritis, intestinal metaplasia, dysplasia, and gastric adenocarcinoma [25].* H. pylori *infection also leads to segregation and chronic stimulation of organized lymphoid tissue. The chronic inflammation of gastric mucosa is dependent on* H. pylori* infection and T cell activity. This inflammation will eventually become autonomous and lead to development of lymphoma [25].* H. pylori *induces MALT lymphoma, the most common lymphoma element in collision tumors [25, 26]. The majority of synchronous MALT-gastric adenocarcinoma tumors present* H. pylori* infection [25].

Another possible carcinogen is Ebstein-Barr Virus (EBV) [15, 26]. EBV infection is known to induce lymphoma and possibly has a strong correlation with gastric adenocarcinoma development. EBV infection leads to delay in apoptosis through upregulation of Bcl-2 and p53 and decrease in cellular differentiation through limited e-cadherin expression [25]. EBV is encountered in 9–16% of gastric adenocarcinoma and 9–16% of lymphoma patients [14].

Finally, there are carcinogenic agents inducing development of tumors of different histological types in the same organ. N-methyl-N-nitro-N-nitrosoguanide induces gastric adenocarcinoma in rats but when combined with agents altering the mucosal barrier it may lead to leiomyosarcoma [18].

The common carcinogen theory is appealing due to the existence of a well-known and established carcinogen,* H. pylori*. This theory explains well synchronous tumors and the predominance of lymphoma-gastric adenocarcinoma coexistence but does not particularly explain the collision phenomenon. It also demands different cell origin of the components at collision neoplasms.

A third hypothesis is the stimulation of tumor-to-tumor carcinogenesis that is one tumor inducing development of a second primary. de Leval et al. [11] described a case of collision of a gastrin-producing carcinoid and a gastric adenocarcinoma. They stated that gastrin's trophic effect on gastric mucosa could induce gastric adenocarcinoma development. Komatsu et al. [27] described a collision tumor of SCC and gastric adenocarcinoma of the gastroesophageal junction. SCC component excreted granulocyte colony stimulating factor (G-CSF) and presented intense lymphoid infiltration. Yanagawa et al. [15] believe that immunosuppression induced by lymphoma could lead to gastric adenocarcinoma development. These hypotheses, while interesting, lack experimental support.

The aforementioned theories assume that collision tumors originate from two different neoplasms and make suggestions on their induction and interaction. However clinical and experimental data indicate that at least some collision tumors arise from a single cell that during tumorigenesis differentiates in two distinct histological types.

Milne et al. [5] performed p53 and loss of heterozygosity (LOH) analysis in two SCC-gastric adenocarcinoma collision tumors of the gastroesophageal junction. They proved that they shared the same p53 mutation and the same LOH pattern. Subsequent comparison of these results with the same analysis in 3 composite tumors (known to consist of two divergent lineages originating from a common precursor cell) showed that 2 out of 3 shared the same p53 mutation and all 3 the same LOH pattern. This constitutes strong evidence that the components of collision tumors originate from the same cell, which differentiates during tumorigenesis maintaining a collision pattern. Secondary mutation, affecting cohesion molecules could be involved but this remains unproven.

Fukui et al. [21] performed the same study on a neuroendocrine carcinoma-gastric adenocarcinoma collision tumor and found the same p53 mutations. Moreover they found different accumulation of p53 at different tumor components and additional p53 mutations at distal parts of the tumor. The tumor studied presented typical histological collision tumor image and different staining of tumor elements at immunohistochemistry.

Finally, while most collision tumors present metastases of one only of their elements, there are reports of lymph node metastases presenting collision patterns [9, 20]. This could indicate that metastases occurred before differentiation of tumor elements yet they differentiated into collision tumors later.

Tumorigenesis of neuroendocrine carcinoma-gastric adenocarcinoma collision tumors can be investigated in a different light. A number of neuroendocrine cells are often present inside gastric adenocarcinomas [28] while neuroendocrine carcinomas are often accompanied by gastric adenocarcinomas in other parts of gastrointestinal track [21]. It has been estimated that a neuroendocrine tumor can coexist with gastric adenocarcinoma in 0.4–4.3% of all cases [29]. Close association of these tumor types has created the concept of Mixed Endocrine Exocrine Carcinomas (MEECs) [30]. They are neoplasms with divergent exocrine and endocrine differentiation with origin in appendix, pancreas, or stomach. For a neoplasm to be characterized as MEEC, it should have at least 30% participation of an endocrine component, although this limit is arbitrary [31]. A common genetic origin of cell components in these neoplasms has been suggested [27, 28]. It is noteworthy that Furlan et al. [32], while studying clonality of a rectal endocrine-exocrine collision tumor, found different origins of the tumor components. MEECs in the stomach are relatively rare; they are more common in the pancreas and appendix [33].

In the literature these neoplasms are often called by different names: composite glandular-endocrine carcinoma, collision tumor, neuroendocrine differentiated gastric adenocarcinoma, amphicrine tumor, and goblet cell carcinoid [33]. MEECs can be categorized into four categories according to their morphological features: carcinomas with interspersed endocrine cells, carcinoids with interspersed nonendocrine cells, amphicrine tumors, and mixed tumors [34]. Mixed tumors can be classified into composite tumors, where histological components are distinct but admixed and present areas of histological transition and collision tumors, where histological components are not admixed and present a clear-cut margin between them [11, 21, 35].

Collision tumors between a neuroendocrine (carcinoid) neoplasm and a gastric adenocarcinoma could represent an extreme form of a well-known and described entity, thus placing collision tumor in the context of a larger group. This theory also enhances the origin of collision tumor from a single cell and is compatible with experimental data. Its disadvantage is that, while supported by genetic studies in neuroendocrine carcinoma-gastric adenocarcinoma and SCC-gastric adenocarcinoma collision tumors, no such data exist for other collision tumor types.

### 2.7. Clinical, Diagnostic, and Therapeutic Implications

As most of the data on collision tumors come from articles with predominately histopathologic interest, relevant clinical information is scarce.

A male predominance in collision tumors has been noted by many authors [14, 36] and is confirmed in this report (46 males and 16 female patients) as is a predominance in the 5th and 6th decade of life. A male predominance has also been noted in synchronous lymphoma and gastric adenocarcinoma patients [25].

Collision tumors are preoperatively indistinguishable from their predominant tumor type and present no differences in terms of clinical and radiological image. Their presenting symptoms are nonspecific and include abdominal pain, loss of appetite, haematemesis, melaena, and weight loss. Additional findings relevant to tumor type are rarely present. Yoshino et al. and de Leval et al. diagnosed such a tumor from high gastrin levels, while Komatsu et al. suspected such a tumor from high G-SF production [11, 27, 37]. Apart from these 2 reports no other specific findings have been described elsewhere. No specific tumor marker rise has been identified either. Finally, abdominal CT and upper GI endoscopy have not been helpful in the detection in the dual nature of the lesion under investigation.

The vast majority of them are diagnosed postoperatively, during histologic examination of gastrectomy specimens. Only rarely they are diagnosed preoperatively [3, 24, 37, 38] with only 2 possible scenarios: either presence of two tumor types in the same sample or different tumor types in two different biopsies. This underlines the importance of multiple tumor biopsy sites.

Data on patient's survival or postoperative therapy are lacking and therefore no meaningful analysis can be performed. In collision lymphoma and gastric adenocarcinoma tumors, prognosis follows that of gastric adenocarcinoma [14]. Most authors believe that the same is true for all collision-type tumors [5, 39, 40]. However, it should be noted that most of these tumors are diagnosed late during their natural course and thus their dismal prognosis might be independent from their particular histological image or collision elements.

It is rational to suggest that surgery remains the cornerstone of the treatment of all resectable nonlymphoma tumors. Adjuvant therapy should target the more advanced or aggressive tumor type [40, 41]. The issue of collision elements being staged independently and treated with separate adjuvant treatment has been discussed [22] but solid evidence is missing. Neoadjuvant treatment has not, to the best of our knowledge, been applied yet either. The issue of individualizing treatment is obviously important in patients with collision tumors, as surgery and systemic treatment must be tailored to meet each patient's needs. These needs may be difficult to define as the two components are at different stage of progress, have different malignant capacity, and respond differently to surgery, radiotherapy, or chemotherapy. The role of the multidisciplinary management is therefore critically important.

## 3. Conclusions

Collision tumors are rare neoplasms consisting of two different histologic patterns and a clear margin with no intermingling. Although rarity of these cases and lack of relevant data preclude detailed investigation, they seem aggressive neoplasms with dismal prognosis. Their origin is obsolete by the time of diagnosis but few available data indicate a single cell origin differentiating into two different histological patterns during tumorigenesis yet maintaining the unique collision pattern between them. Their relationship with composite and amphicrine tumors is not clear but they share common characteristics. Clinically they are usually indistinguishable from the dominant tumor type and diagnosis is almost always set postoperatively on histology. Despite their rarity, their unique characteristics could shed light on many aspects of tumorigenesis including differentiation and adhesion. Surgeons and pathologists should be aware of those rare entities and when encountered should be submitted to proper histologic and molecular analysis.

## Figures and Tables

**Table 1:** Neuroendocrine carcinoma and gastric adenocarcinoma collision tumors.

Author	Sex/age	Gastric adenocarcinoma	Nodes	Follow-up	Adjuvant treatment
Grossi and Lattes [39], 1956	M/74	N/A	N/A	12 m	N/A
Parks [20], 1970	M/54	N/A	NEC/GA (+)	3 m (DOD)	N/A
Yamashina and Flinner [40], 1985	M/50	Mixed	NEC (+)	N/A	N/A
Yoshino et al. [37], 1987	F/63	WD	N/A	N/A	N/A
Chodankar et al. [41], 1989	F/69	WD	—	N/A	N/A
Morishita et al. [28], 1991	M/49	MD	N/A	N/A	N/A
Corsi and Bosman [42], 1995	M/72	MD	N/A	N/A	N/A
Fukui et al. [21], 2001	M/63	PD	N/A	N/A	N/A
de Leval et al. [11], 2002	M/87	WD	NEC (+)	1 m (DOD)	N/A
Olinici et al. [43], 2004	M/68	N/A	N/A	N/A	N/A
Morishita et al. [44], 2005	F/84	MD	GA (+)	33 m	N/A
Jayaraman et al. [45], 2005	M/48	MD	N/A	N/A	N/A
Jang et al. [10], 2010	M/50	WD	NEC (+)	N/A	N/A
Doggui et al. [12], 2008	M/55	N/A	N/A	3 m (DOD)	Chemo
Mardi et al. [46], 2008	F/47	WD	GA (+)	N/A	N/A
Mróz et al. [8], 2009	M/56	PD	GA (+)	N/A	N/A
Lee et al. [33], 2011	M/62	PD	—	N/A	N/A
Ünal et al. [35], 2013	F/51	MD	GA (+)	5 m (DOD)	N/A

N/A: Not available.

WD: Well differentiated.

MD: Moderately differentiated.

PD: Poorly differentiated.

NEC: Neuroendocrine carcinoma.

GA: Gastric adenocarcinoma.

m: Months.

DOD: Dead of disease.

**Table 2:** Lymphoma and gastric adenocarcinoma collision tumors.

Author	Sex/age	Lymphoma	Gastric adenocarcinoma	Nodes	Follow-up	Adjuvant treatment
Jernstrom and Murray [9], 1966	F/72	Lymphosarcoma	WD, I	L/GA (+)	10 m	Radiotherapy

Manier and Reyes [3], 1974	M/65	Histiocytic	EGC, PD	L (+)	24 m	Chemotherapy
	M/72	Histiocytic	EGC, PD	L/GA (+)	1,5 m (DOS)	None

Planker et al. [47], 1984	M/65	Immunocytoma	EGC, MD, I	L (+)	N/A	Radiotherapy

Kasahara et al. [4], 1988	F/72	Small cleaved B cells	WD	(−)	48 m	N/A

Noda et al. [38], 1989	M/69	Large-cell type	EGC, WD	(−)	9 m	Chemotherapy

Wotherspoon and Isaacson [24], 1995	F/55	MALT	WD, D	GA (+)	N/A	N/A
	F/55	MALT	AGC, WD, I	GA (+)	N/A	N/A
	M/N/A	MALT	AGC, WD, I	(−)	N/A	N/A
	F/69	MALT	AGC, WD, I	L/GA (+)	N/A	N/A

Nishino et al. [13], 1996	M/71	Diffuse large cell	WD	(−)	120 m	Chemotherapy

Nakamura et al. [14], 1997	M/42	Immunoblastic	EGC, PD, D	(−)	91 m	None
	M/47	Superficial MALT	EGC, PD, D	(−)	24 m	Chemotherapy
	M/53	Superficial MALT	EGC, WD, I	(−)	67 m (DOD)	None
	F/67	Superficial MALT	EGC, WD, I	(−)	31 m (DOD)	None
	M/78	T cell, pleomorphic	AGC, PD, D	GA (+)	1 m (DOS)	None

Goteri et al. [6], 1997	M/51	MALT	EGC, WD, I	(−)	122 m	N/A
	F/55	MALT	EGC, PD, D	(−)	33 m	N/A
	M/80	MALT	EGC, WD, I	L (+)	12 m	N/A
	M/57	MALT	EGC, WD, I	L (+)	10 m (DOD)	N/A

Suenaga et al. [23], 2003	M/73	MALT	AGC, WD, I	GA (+)	23 m (DOD)	N/A

Isaka et al. [48], 2007	F/63	MALT	N/A	N/A	2 m (DOD)	N/A

Bhattacharya et al. [29], 2012	M/55	NHL	WD	N/A	N/A	N/A
	M/67	NHL	N/A	N/A	N/A	N/A

Yanagawa et al. [15], 2012	M/72	HL	PD	L (+)	N/A	N/A

George and Junaid [49], 2014	M/53	MALT	SRC	N/A	2 m	N/A

MALT: Mucosa associated lymphoid tissue.

NHL: Non-Hodgkin's lymphoma.

HL: Hodgkin's lymphoma.

WD: Well differentiated.

MD: Moderately differentiated.

PD: Poorly differentiated.

EGC: Early gastric cancer.

AGC: Advanced gastric cancer.

D: Diffuse.

I: Intestinal.

L: Lymphoma.

GA: Gastric adenocarcinoma.

SRC: Signet ring cell.

m: Months.

DOD: Dead of disease.

N/A: Not available.

**Table 3:** Squamous cell carcinoma and gastric adenocarcinoma collision tumors.

Author	Sex/age	SCC	GA	Nodes	Follow-up	Adjuvant treatment
Wanke [50], 1972	M/52	N/A	N/A	(−)	N/A	N/A
Majmudar et al. [7], 1978	M/63	WD	N/A	GA (+)	N/A	N/A
Spagnolo and Heenan [51], 1980	M/73	WD	MD	N/A	2 m (DOD)	—
	M/50	MD	MD	N/A	Not operated	
Washizawa et al. [52], 1999	M/68	WD	MD	SCC/GA (+)	12 m (DOD)	N/A
Komatsu et al. [27], 2003	M/73	MD	WD	SCC (+)	19 m	N/A
Milne et al. [5], 2004	N/A	N/A	N/A	N/A	N/A	N/A
	N/A	N/A	N/A	N/A	N/A	N/A
Santos et al. [53], 2006	M/37	MD	MD	(−)	11 m (DOD)	N/A

SCC: Squamous cell carcinoma.

GA: Gastric adenocarcinoma.

WD: Well differentiated.

MD: Moderately differentiated.

m: Months.

DOD: Dead of disease.

N/A: Not available.

**Table 4:** Gastrointestinal stromal tumor and gastric adenocarcinoma collision tumors.

Author	Sex/age	GIST	GA	Nodes	Follow-up	Adjuvant treatment
Liu et al. [54], 2002	M/70	0/50 hpf	AGC, I	GA (+)	3 m (DOAC)	None
Katsoulis et al. [55], 2007	F/78	N/A	AGC, PD, D	GA (+)	N/A	N/A
Idema et al. [56], 2008	M/71	<5/50 hpf	AGC, D	GA (+)	N/A	N/A
Trabelsi et al. [57], 2008	M/54	0/50 hpf	AGC, SRC	GA (+)	N/A	N/A
Bi et al. [58], 2009	F/73	5/50 hpf	WD, I	GA (+)	N/A	N/A
Kleist et al. [16], 2010	F/86	<5/50 hpf	WD, I	(−)	11 m	N/A
	M/78	<5/50 hpf	PD, SRC	(−)	4 m (DOD)	N/A

GIST: Gastrointestinal stromal tumor.

GA: Gastric adenocarcinoma.

Hpf: High power field.

AGC: Advanced gastric cancer.

D: Diffuse.

I: Intestinal.

SRC: Signet ring cell.

PD: Poorly differentiated.

m: Months.

DOD: Dead of disease.

DOAC: Dead of another cause.

N/A: Not available.
